# Loss of Renal Allografts Secondary to Candida Vascular Complications in Two Recipients from the Same Donor

**DOI:** 10.1155/2012/364735

**Published:** 2012-03-07

**Authors:** Govardhana Rao Yannam, Lucile Wrenshall, R. Brian Stevens

**Affiliations:** Transplant Surgery Division, Department of Surgery, University of Nebraska Medical Center, Omaha, NE 68198-3285, USA

## Abstract

Infections remain a major cause of morbidity and mortality in transplant patients. Organ recipients are also susceptible to donor-derived pathogens and the majority of donor infections are easily treatable. Rarely, some pathogens have produced life-threatening complications by compromising the vascular anastomosis. In this case series we report loss of two kidney allografts secondary to vascular complications due to Candida albicans. Both recipients received grafts from a common donor, in whom Candida bacteremia in the donor was not apparent at the time of organ acceptance but became apparent on delayed cultures.

## 1. Introduction

Infections remain a major cause of morbidity and mortality in solid organ transplant recipients. In addition to nosocomial infections, transplant patients are also susceptible to a variety of donor-derived pathogens [1]. Fortunately, the majority of donor pathogens are commensal bacterial flora with little pathogenic potential. Occasionally pathogens such as Staph aureus, gram-negative bacilli, anaerobic bacteria, *Candida albicans*, and Aspergillus have produced life-threatening complications in organ recipients by compromising the vascular anastomosis [2, 3]. Candida vasculitis is a rare devastating complication of kidney transplantation. Here we report on two organ recipients lost their grafts due to Candida infection presumably transmitted from a common donor.

## 2. Case History

The donor was a 21-year-old man, a victim of motor vehicle accident, underwent initial resuscitation and intubation at accident site, and hospitalized 4 days prior to declaration of brain death and multiorgan procurement. Routine lab tests were normal including blood and urine cultures. Sputum culture was positive for *Candida albicans*. Liver and both kidneys were harvested without incident. We do not add any antibiotics to the preservative solution according to our department policy.

The 1st recipient was a 51-year-old Filipino-American female with end-stage renal disease (ESRD) secondary to focal segmental glomerulosclerosis. Patient was induced with thymoglobulin and steroids, maintained on tacrolimus and mycophenolate mofetil (MMF) (steroid free), and had good graft function. On postoperative day (POD) 17, he presented to the ER with hypotension and low hematocrit. A CT scan revealed a large perinephric hematoma and underwent emergent exploration. An arterial bleeder at the renal hilum was ligated with restoration of hemostasis. On POD29 the patient presented with acute abdomen and pulsatile mass across the lower abdomen. On exploration complete disruption of the arterial anastomosis with necrosis of the renal artery and adjacent external iliac artery was noted. Explant of the renal graft with resection of the necrotic segments of iliac artery and primary repair was done. Histological examination did not show any evidence of fungus, but a blood clot sent intraoperatively grew *Candida albicans. *With antifungal therapy, patient had protracted recovery.

**Figure 1:**
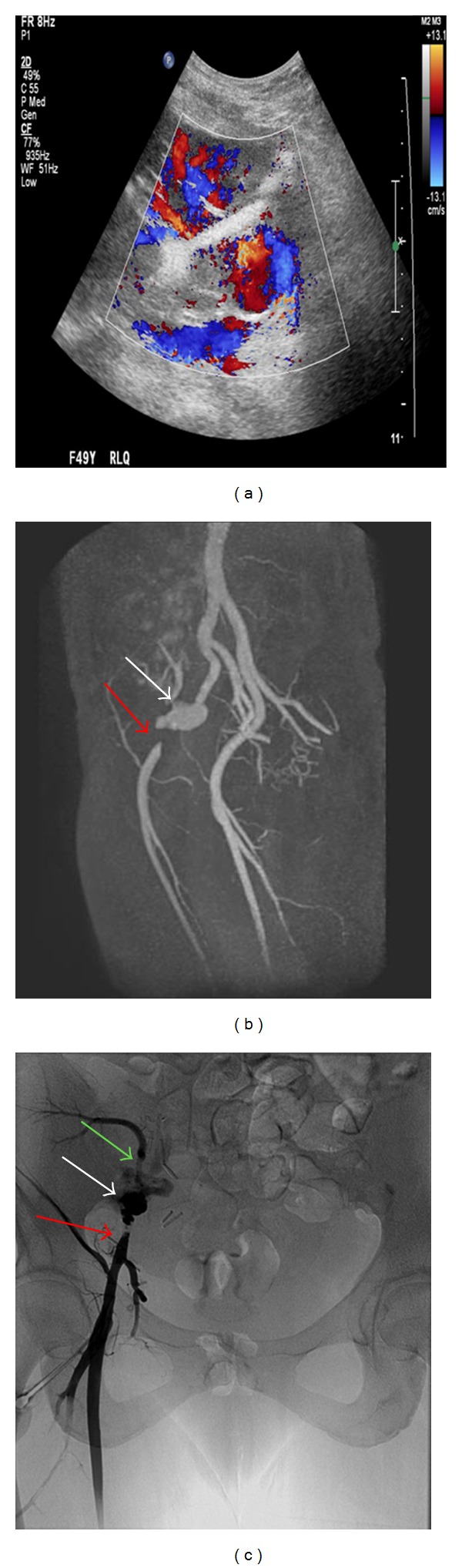
A possible pseudoaneurysm in the region of the vascular anastomosis revealed by Color Doppler Ultrasound (a) with an area of turbulent and to-and-fro waveforms. Follow-up MRI (b) confirmed a bilobed pseudoaneurysm (white arrow) and revealed a segment of high-grade stenosis of the right external iliac artery (red arrow). Conventional angiogram image (c) in AP view reconfirmed the pseudoaneurysm (white arrow) and a segment of high-grade stenosis of the right external iliac artery (red arrow) with stenosis of the transplanted renal artery (green arrow).

**Figure 2:**
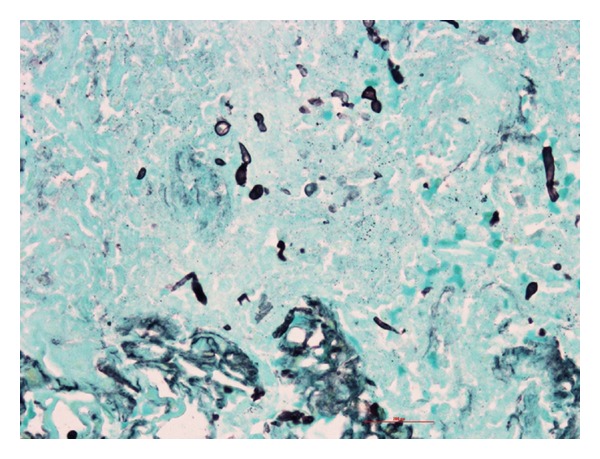
Pathological examination of explant revealed renal artery and intraparenchymal arterial branches positive for fungal organisms by GMS stain.

The 2nd recipient was a 49-year-old Hispanic female with ESRD secondary to glomerulosclerosis. She received a similar induction, followed by tacrolimus and rapamycin maintenance (steroid-free), and had good graft function. She was admitted on POD88 with febrile neutropenia and painful mouth sores. Creatinine remained normal and blood and urine cultures were negative. Neutropenia responded quickly to Neupogen, fevers resolved, and patient was discharged 4 days later. In view of neutropenia and oral lesions, rapamycin was substituted with MMF. She was readmitted with fevers and elevated creatinine on POD112. Imaging studies performed are shown in Figure 1. Patient had semiurgent exploration and attempts to salvage the graft were unsuccessful. The patient underwent nephrectomy and resection of a portion of the external iliac artery with reconstruction with reversed saphenous vein graft. Subsequent blood cultures grew *Candida albicans*. Histopathology of explant is shown in Figure 2. An echo was negative for valvular vegetations. With antifungal therapy for 8 weeks, patient recovered uneventfully.

In retrospect Candida bacteremia in the donor was not apparent at the time of organ acceptance but became apparent on delayed cultures, but not available for patient management. We could have investigated for potential Candida infection in the 2nd recipient soon after the first case to prevent such an outcome. Transplant teams should be vigilant and follow other organ recipients from the same donor when donor transmitted infection is a possibility.

## 3. Discussion

Donor-derived infections may occur at different phases of the transplantation: (a) from donor, especially from victims of polytrauma and in those who had prolonged intensive care unit stay; (b) during harvest, particularly multiorgan procurement, injury to gut during procurement, and harvesting kidneys from non-heart-beating donors; and (c) during organ preservation and graft implantation (contamination of preservation fluid) [4].

Vascular Candidal infections typically present in 2 forms.

1. Anastomotic leak or vessel rupture: it occurs early in the postoperative course (within 2 months post-transplant) and responsible for 2/3 of cases [4]. Lack of antecedent symptoms makes early diagnosis difficult. Patients usually present with massive bleeding requiring emergency surgical intervention. The mycotic arteritis typically involves the renal artery along with adjacent iliac artery. Attempts of vascular repair are usually unsuccessful, that was evident in our first case. Most patients end up with transplant nephrectomy along with resection of adjacent portion of recipient vessel. It is advisable to avoid placing prosthetic material to reestablish vascular continuity for fear of recurrent infections.
2. Pseudoaneurysm: it occurs “late” after transplant (>2 months) [4]. With appropriate diagnosis and planning, one can attempt to salvage the allograft. Though our second patient presented to us 3 months after surgery with febrile neutropenia, presence of severe oral ulcers and elevated rapamycin levels prompted us to switch over to MMF. Stopping rapamycin, rapid response of fevers with antibacterials, and normal creatinine did not warrant any further imaging. Subsequently elevated creatinine prompted us to image transplant kidney and led to the diagnosis. To date, in only 5 of 18 reported cases (including the present report) presented in this fashion, and in only three, the allografts were salvaged. Rapid evaluation of organ-donor suitability is essential in transplantation. Information regarding donor pathogens particularly about slow-growing species such as fungi will often not be available at the time organ allocation. Therefore, tests used to screen potential donors for transmissible infections must be rapid, sensitive, reproducible, and readily available to organ procurement organizations. Since usage of organs from a single donor to different recipients in different transplantation centers is common, an immediate system for tracking and disseminating pertinent data needs to be established.

Another important preventive measure may be routine culture of preservation media to detect contamination during organ procurement and/or graft back-table preparation. The rate of positive culture of preservation fluid ranged from 5 to 23% and fungi represented 2 to 10% of all positive cultures [5]. It is recommended that cultures be routinely maintained for extended periods to detect late-growing pathogens. Positive preservation fluid cultures are sometimes the only clue for the evidence of fungal infection. Starting appropriate antibiotic and antimycotic therapy depending on positive cultures from the preservation fluid may prevent vascular complications. The utility of this therapy is unknown at present.

In conclusion, mycotic arteritis due to *C. albicans *is a potentially life-threatening complication of renal transplantation leading to graft loss in most of the cases and utmost care is needed to prevent contamination at all phases of transplantation to prevent this complications. It is of utmost importance to report and follow up other organ recipients from the same donor if one encounters a suspected donor-related infection to prevent devastating complications.
